# Modulation of innate immune responses at birth by prenatal malaria exposure and association with malaria risk during the first year of life

**DOI:** 10.1186/s12916-018-1187-3

**Published:** 2018-11-02

**Authors:** Hamtandi Magloire Natama, Gemma Moncunill, Eduard Rovira-Vallbona, Héctor Sanz, Hermann Sorgho, Ruth Aguilar, Maminata Coulibaly-Traoré, M. Athanase Somé, Susana Scott, Innocent Valéa, Petra F. Mens, Henk D. F. H. Schallig, Luc Kestens, Halidou Tinto, Carlota Dobaño, Anna Rosanas-Urgell

**Affiliations:** 1 Department of Biomedical Sciences, Institute of Tropical Medicine, B 2000 Antwerp, Belgium; 2 Unité de Recherche Clinique de Nanoro, Institut de Recherche en Sciences de la Santé, BP218, Nanoro, Burkina Faso; 3 Department of Biomedical Sciences, University of Antwerp, B 2610 Antwerp, Belgium; 4 Barcelona Institute for Global Health (ISGlobal), Hospital Clinic – Universitat de Barcelona, Carrer Rossello 132, E-08036 Barcelona, Catalonia Spain; 5 Department of Epidemiology and Population Health, London School of Hygiene and Tropical Medicine, London, WC1E7HT UK; 6 Department of Medical Microbiology - Parasitology Unit, Academic Medical Centre, Amsterdam, 1105 AZ The Netherlands; 7 Centre Muraz, BP390, Bobo Dioulasso, Burkina Faso

**Keywords:** Malaria in pregnancy, Prenatal malaria exposure, Innate immunity, Cytokines, Toll-like receptor, Malaria in infancy

## Abstract

**Background:**

Factors driving inter-individual differences in immune responses upon different types of prenatal malaria exposure (PME) and subsequent risk of malaria in infancy remain poorly understood. In this study, we examined the impact of four types of PME (i.e., maternal peripheral infection and placental acute, chronic, and past infections) on both spontaneous and toll-like receptors (TLRs)-mediated cytokine production in cord blood and how these innate immune responses modulate the risk of malaria during the first year of life.

**Methods:**

We conducted a birth cohort study of 313 mother-child pairs nested within the COSMIC clinical trial (NCT01941264), which was assessing malaria preventive interventions during pregnancy in Burkina Faso. Malaria infections during pregnancy and infants’ clinical malaria episodes detected during the first year of life were recorded. Supernatant concentrations of 30 cytokines, chemokines, and growth factors induced by stimulation of cord blood with agonists of TLRs 3, 7/8, and 9 were measured by quantitative suspension array technology. Crude concentrations and ratios of TLR-mediated cytokine responses relative to background control were analyzed.

**Results:**

Spontaneous production of innate immune biomarkers was significantly reduced in cord blood of infants exposed to malaria, with variation among PME groups, as compared to those from the non-exposed control group. However, following TLR7/8 stimulation, which showed higher induction of cytokines/chemokines/growth factors than TLRs 3 and 9, cord blood cells of infants with evidence of past placental malaria were hyper-responsive in comparison to those of infants not-exposed. In addition, certain biomarkers, which levels were significantly modified depending on the PME category, were independent predictors of either malaria risk (GM-CSF TLR7/8 crude) or protection (IL-12 TLR7/8 ratio and IP-10 TLR3 crude, IL-1RA TLR7/8 ratio) during the first year of life.

**Conclusions:**

These findings indicate that past placental malaria has a profound effect on fetal immune system and that the differential alterations of innate immune responses by PME categories might drive heterogeneity between individuals to clinical malaria susceptibility during the first year of life.

**Electronic supplementary material:**

The online version of this article (10.1186/s12916-018-1187-3) contains supplementary material, which is available to authorized users.

## Background

Despite the widespread implementation of Intermittent Preventive Treatment with sulfadoxine-pyrimethamine (IPTp-SP) to prevent malaria during pregnancy, infants in endemic countries are often born to mothers with placental malaria (PM). This is likely to increase their risk of a malaria infection in early childhood [1–7]. Factors explaining the association between PM and risk of malaria infection during infancy are still not well understood, but this association has been correlated with changes in malaria-specific fetal immunity [8]. Cord blood mononuclear cells of neonates born to mothers with PM can specifically respond to plasmodial asexual blood stage antigens, impacting on immune response to *Plasmodium falciparum* infection during infancy [9–15]. This prenatal exposure to malaria-infected erythrocytes or their soluble products can lead to fetal immune priming to malaria blood stage antigens or to fetal immune tolerance in some infants [11, 16–20]. Nonetheless, factors that lead to this inter-individual difference in immune responses to malaria antigens upon prenatal exposure are unknown.

In early infancy, innate immunity is the main defense barrier of the host, as newborns have a naïve adaptive immune system [21, 22]. The immune cellular response starts with the recognition of pathogen molecules known as pathogen-associated-molecular patterns (PAMPs) by cells of the innate immune system through pattern recognition receptors (PRRs). Among these receptors, it has been shown that toll-like receptors (TLRs) are key initiators of innate immunity and promoters of adaptive immunity via direct and indirect mechanisms [23–25]. Ligands binding to TLRs generate intracellular signals, activate gene expression, and enhance the release of cytokines and chemokines [26, 27], which are important players in the pathogenesis of and protection against malaria [28]. Therefore, in early life, protection from infections relies extensively on innate immunity, and hence, factors that modulate the development of fetal innate immunity may drive variation in susceptibility to malaria between individuals in early infancy.

A few studies have reported that history of *P. falciparum* infections during pregnancy may have an effect on neonatal innate immune responses upon TLRs stimulation with implications for the outcome of newly encountered infections in early life [11, 29, 30]. Cytokine responses upon TLRs stimulation of cord blood cells have been found to be profoundly affected by either maternal peripheral infections occurring late in pregnancy [29, 30] or past PM [11]. In addition, it has been shown that exposure to malarial antigens in utero has different effects on the immune environment at birth, such as the number and/or activation status of immune cell populations, including antigen-presenting cells, regulatory, and effector CD4^+^ T cells, depending on the type of exposure [10–15]. Overall, these data indicate that maternal peripheral and placental infections during pregnancy have an impact on cord blood cytokine responses to TLR agonists and that time and type of malaria exposure can skew cytokine responses towards a regulatory/tolerogenic or to a proinflammatory profile. In this regard, a tolerogenic profile would render infants more susceptible to malaria infections during the first year of life, whereas a proinflammatory profile can lead to more severe malaria episodes, whereas a Th1/Th17 profile could be protective.

Human TLRs that are known to be stimulated by malaria parasite-derived molecules include TLR2 (by glycosylphosphatidylinositol), TLR4 (by hemozoin), and TLR9 (by hemozoin and parasite DNA) [31–34]. However, the clinical relevance of TLR-mediated immune responses in the susceptibility to malaria has been mainly reported for endosomal PRRs such as TLR3, TLR7/8, and TLR9 in African children. Indeed, higher TLR3- and TLR7/8-mediated interleukin (IL)-10 responses at birth were found to be associated with a significant increased risk of *P. falciparum* infection in infants in Benin [30], whereas polymorphisms in TLR9 gene were associated with difference in susceptibility to malaria in Burundian and Ghanaian children [35, 36].

In this study, we assessed the effect of different types of prenatal malaria exposure (PME) on endosomal TLR-mediated cytokine responses in cord blood samples collected at birth, and we investigated the subsequent risk of malaria during the first year of life in a highly seasonal malaria endemic area of Burkina Faso.

## Methods

### Study design and participants

**Fig. 1:**
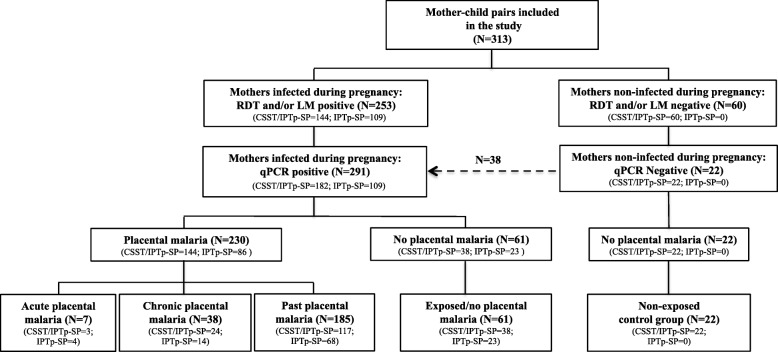
Categories of prenatal malaria exposure (PME). Pregnant women infected during pregnancy with placental malaria (PM; acute, chronic, or past) or without PM (exposed/no PM) were recruited from both COSMIC study arms [37]. Pregnant women included in the non-exposed control group were only recruited among the CSST/IPTp-SP intervention arm: all of them had negative RDT/LM results for malaria infection in monthly screenings and at antenatal care visits that were later on confirmed by qPCR and, with no evidence of placental malaria

A prospective birth cohort study was nested within the COSMIC trial (NCT01941264). In brief, COSMIC was a cluster randomized controlled trial investigating the protective efficacy of adding community-scheduled screening and treatment of malaria during pregnancy (CSST) to the standard IPTp-SP (CSST/IPTp-SP; intervention arm) compared to IPTp-SP alone (control arm) in Burkina Faso, Benin, and The Gambia [37]. The CSST extension strategy was implemented through monthly screening using rapid diagnostic tests (RDTs) and treatment of malaria infections with artemether-lumefantrine (AL). Pregnant women in both arms who experienced clinical malaria during pregnancy were also treated with AL. In addition, all pregnant women in the two arms were further screened for malaria during antenatal care (ANC) booking using light microscopy (LM). Furthermore, additional blood spots on filter papers during community screening (CSST/IPTp-SP arm) and at each ANC visit (both CSST/IPTp-SP and IPTp-SP arms) were collected for posterior malaria diagnostic by quantitative real-time polymerase chain reaction (qPCR). At the time of delivery, placenta biopsies and cord blood samples in heparin containing tubes were collected from mother-child pairs. Placenta histology was performed later on within the parent COSMIC trial, while cord blood samples were immediately processed. Of the 734 mother-child pairs enrolled in the birth cohort in Burkina Faso, a subgroup of 313 mothers and their offspring were included for the present study. Those mother-child pairs were selected based on the history of malaria infection during pregnancy (using LM and RDT results) and the availability of cord blood samples for immunological assays at delivery (Fig. 1). The study was conducted in the rural health district of Nanoro, a high and seasonal malaria transmission area in Centre-West of Burkina Faso [38].

### Recruitment and follow-up

The recruitment procedure of the mother-child pairs and details of the 1-year follow-up of infants included in the birth cohort study have been previously described [39, 40]. Shortly, pregnant women from Nanoro participating in the COSMIC trial were asked at antenatal care visits to participate in the birth cohort study prior to delivery. At delivery, healthy newborns with their mothers were enrolled after informed consent was obtained. Exclusion criteria were presence of major congenital malformation, chronic disease, or signs of cerebral asphyxia. Clinical malaria episodes in infants were monitored by passive case detection, for which mothers were encouraged to seek care in peripheral health centers at any time their child felt sick. At each attendance to health facilities, a clinical examination was performed and mothers were asked for previous health events. In the case of fever (axillary temperature ≥ 37.5 °C) or history of fever in the previous 24 h, a malaria RDT was performed and positive infants were treated according to national guidelines.

### Sample collection

Sample collection procedures have been described elsewhere [41]. In brief, at time of delivery, approximately 200 μL of maternal peripheral blood was obtained by finger-prick for blood smear preparation and blood spot on filter paper. A placental tissue section was collected from the maternal side and preserved in 10% neutral buffer formalin at 4 °C for histology examination. In addition, cord blood (approximately 10 mL) was collected in heparin-containing tubes by venipuncture of the umbilical vein for TLRs stimulation assays. The remaining cord blood in the heparinized tube was transferred from the peripheral health centers to the laboratory at the Clinical Research Unit of Nanoro (CRUN) for processing within 4 h. Peripheral blood was collected post-partum by finger-prick from each infant visiting the health facilities with presence of fever or history of fever in the previous 24 h, and used for RDT, blood smear, and spots on filter paper (Whatman 3MM).

### Toll-like receptors stimulation assay

TLRs stimulations of cord blood mononuclear cells were performed using fresh whole cord blood samples. Briefly, cord blood samples were diluted 1:1 with RPMI 1640 (1X, Gibco) and five aliquots of 200 μl were prepared. One aliquot was left unstimulated and the other four were stimulated either with the synthetic analog of dsRNA-PolyI:C (TLR3 ligand; 10 μg/mL; InvivoGen, San Diego, USA), imidazoquinoline (R848, TLR7/8 ligand; 10 μg/mL; InvivoGen, San Diego, USA), the synthetic type B unmethylated CpG dinucleotide (ODN2006-1, TLR9 ligand, 5 μM; InvivoGen, San Diego, USA) or with a mixture of phorbol myristate acetate (PMA), and ionomycin as positive control (PMA 0.1 μg/mL and ionomycin 1 μg/mL; Sigma-Aldrich, Schnelldorf, Germany). After 24 h of incubation at 37 °C in 5% CO_2_, supernatants were collected following a centrifugation at 500*g* for 5 min, then frozen at − 80 °C. Culture supernatants were subsequently shipped frozen to ISGlobal (Barcelona) for cytokines, chemokines, and growth factors measurement.

### Cytokines, chemokines, and growth factors quantification

Supernatants were thawed at room temperature, centrifuged at 1000*g* for 10 min and then diluted in a ratio of 1:5 in RPMI 1640 (1X, Gibco). Cytokines, chemokines, and growth factors levels were determined using a fluorescent bead-based multiplex immunoassay (Human Cytokine Magnetic 30-Plex Panel kits, Novex®, Life Technologies™, USA). Twenty-five microliters of each supernatant were tested in single replicates applying a modification of the manufacturer’s protocol, which implies using half the volume of each reagent except for the washing buffer. The 30-Plex panel kit includes the following: interleukin (IL)-2, IL-4, IL-5, IL-6, IL-7, IL-8, IL-10, IL-13, IL-15, IL-17, IL-1β, IL-1RA, IL-2R, IL-12(p40/p70), tumor necrosis factor (TNF), interferon (IFN)-γ, IFN-α, IFN-γ inducible protein 10 (IP-10), monocyte chemottractant protein (MCP)-1, macrophage inflammatory protein (MIP)-1α, MIP-1β, eotaxin, RANTES, monokine-induced by IFN-γ (MIG), vascular endothelial growth factor (VEGF), hepatocyte growth factor (HGF), epidermal growth factor (EGF), fibroblast growth factor (FGF) basic, granulocyte-colony stimulating factor (G-CSF), and granulocyte-macrophage colony stimulating factor (GM-CSF). Samples were acquired on a Luminex® 100/200™ instrument using Xponent 3.1 software. Median fluorescent intensity (MFI) data was analyzed using the drLumi 0.1.2 R package [42], in which concentration of each analyte was determined by interpolating the MFI to a standard curve (plotted using a 5- or 4-parameter logistic function) of twofold 16 serial dilutions prepared from a reference sample provided by the manufacturer. The limits of quantification (lower, LLOQ and upper, ULOQ) for each analyte and plate were obtained applying the 20% coefficient of variation method [43–45] in drLumi. Any analyte with a value below the LLOQ was given a value of half the LLOQ for that analyte, and any analyte with a value above the ULOQ was given a value of two times the ULOQ of that analyte.

### Malaria detection and definitions

SD-Bioline malaria antigen P.f® test (05FK50, Standard Diagnostics, Inc., Korea) detecting PfHRP2 was used for malaria RDT according to the manufacturer’s instructions. The microscopic examination of thick blood smears stained with Giemsa (10%) was performed according to standard procedures [46]. Dried blood spots on filter paper were used for DNA extraction (QIAamp 96 DNA blood kit, Qiagen, Germany) and, *P. falciparum* detection of *Pf*-varATS by qPCR, as previously described [41]. Data on past history of malaria infections during pregnancy and histological examination of placental tissues were obtained from the COSMIC trial [37]. A clinical malaria episode was defined as the detection of *P. falciparum* parasites by qPCR and presence of fever. PM infections were defined by histological examination as follows: (i) acute infection (parasites present, malaria pigment absent), (ii) chronic infection (parasites and malaria pigment present), (iii) past infection (parasites absent but pigment present), and (iv) no infection (both parasites and malaria pigment absent). PME was categorized based on placental infection (past, chronic, acute) and maternal peripheral infection as shown in Fig. 1. The non-exposed control group was composed of pregnant women only recruited among the CSST/IPTp-SP intervention arm who had negative RDT/LM and qPCR results at each screening and ANC visit and negative placental histology.

### Statistical analysis

Statistical analysis was performed using R statistical package version 3.2.3 [47]. Cytokine concentrations (both crude and ratios between stimulated and unstimulated samples) were log_10_-transformed after assessing the distribution of each cytokine using normality plots for each cytokine across TLR stimulations. To explore sample clusters by TLR stimulation, data were plotted by using principal component analysis (PCA) and the first two components were used to show associations.

To assess the effect of PME on TLR-mediated cytokine responses, ANOVA test was used to compare the mean of cytokine responses between groups of PME for significant variance among the mean of cytokine responses. Benjamini-Hochberg method was applied to adjust *p* values for multiple comparisons [48]. Maternal- and infant-related co-variables including gravidity, low birth weight (LBW), birth season, newborn sex, and ethnicity were used to adjust the effect of PME on cytokine responses in linear regression models.

The association between TLR-mediated cytokine responses at birth and the risk of clinical malaria during the first year of life was assessed in univariable and multivariable Cox proportional-hazard models. Proportionality of hazards assumption and functional form of each variable adjusted in the Cox models was examined using Schoenfeld residuals analysis and p-splines, respectively. Secondary variables that showed significant association with malaria during the first 12 months of life were determined in Kaplan-Meier survival analyses (log-rank test *P* value < 0.05) and included in the Cox proportional-hazard regression models. A *P* value < 0.05 was considered statistically significant.

## Results

### Characteristics of study participants

The characteristics of the participants included in this study are presented in Table 1. The mean age of pregnant women at enrollment was 26.1 years, and the majority of them were multigravida (63%). More than two thirds of deliveries (77.6%) occurred during the malaria high-transmission season (July–December). The mean birth weight of the newborns was 3009 g, while 9.6% had a low birth weight (LBW). In total, 291 newborns (93%) were exposed to malaria parasites and/or antigens in utero. The majority of the newborns were born to mothers with past PM (59.1% [185/313]) followed by those born to mothers who had either clinical malaria (*N* = 6) or asymptomatic infection (*N* = 55) during pregnancy but with no evidence of PM at delivery (19.5% [61/313]). Few infants were born to mothers with acute PM at delivery (2.2% [7/313]). There was a higher but non-significant proportion of females than males among the newborns (*P* = 0.158).

**Table 1:** Characteristics of study participants

Characteristics	Overall cohort (*N* = 313)	Non-exposed (*N* = 22)	Exposed no PM (*N* = 61)	Past PM (*N* = 185)	Chronic PM (*N* = 38)	Acute PM (*N* = 7)	*P* value
Maternal characteristics							
Age (years, mean ± SD)	26.1 ± 6.2	28.4 ± 6.4	27.7 ± 6.0	25.5 ± 6.0	23.8 ± 5.7	28.3 ± 7.2	< 0.001
Gravidity (*N* (%))							< 0.001
Primigravida	58 (18.5)	1 (4.5)	2 (3.3)	40 (21.6)	15 (39.5)	0 (0.0)	
Secundigravida	58 (18.5)	2 (9.1)	10 (16.4)	38 (20.5)	7 (18.4)	1 (14.3)	
Multigravida	197 (63.0)	19 (86.4)	49 (80.3)	107 (57.9)	16 (42.1)	6 (85.7)	
ITN use (*N* (%))	219 (70.0)	19 (86.4)	47 (77.0)	128 (69.2)	21 (55.3)	4 (57.1)	0.061
MiP preventive strategy in COSMIC trial (N (%))							< 0.001
Standard IPTp-SP	109 (34.2)	–	23 (37.7)	68 (36.8)	14 (36.8)	4 (57.1)	
CSST/IPTp-SP	204 (65.2)	22 (100.0)	38 (62.3)	117 (63.2)	24 (63.2)	3 (42.9)	
SP doses uptake (women who received ≥ 2 doses, *N* (%))	293 (93.6)	21 (95.5)	55 (90.2)	178 (96.2)	32 (84.2)	7 (100.0)	0.055
AL treatment (women treated at least once, *N* (%))	67 (21.4)	–	7 (11.5)	43 (23.2)	14 (36.8)	3 (42.8)	0.002
Gestational age at enrollment (median (IQR), weeks)	20 (19–22)	20 (18–26.5)	20 (20.5–25.5)	20 (19–21)	20 (16–21)	20 (20–25)	0.445
Infants characteristics							
Sex (females, *N* (%))	169 (54.0)	7 (31.8)	36 (59.0)	105 (56.8)	19 (50.0)	2 (28.6)	0.110
Birth season (malaria high-transmission season, *N* (%))	243 (77.6)	14 (63.6)	44 (72.1)	141 (76.2)	37 (97.4)	7 (100.0)	0.002
Birth weight (g, mean ± SD)	3009 ± 429.6	3119.1 ± 441.7	3041.5 ± 360.5	2988.2 ± 439.1	2967.1 ± 499.8	3169.3 ± 228.4	0.470
LBW (< 2500 g) (no. (%))	30 (9.6)	1 (4.5)	3 (4.9)	20 (10.8)	6 (15.8)	0 (0.0)	0.371
Ethnicity (*N* (%)							0.017
Mossi	276 (88.2)	21 (95.4)	55 (90.1)	164 (88.6)	31 (81.6)	5 (71.4)	
Gourounsi	34 (10.8)	1 (4.6)	4 (6.6)	21 (11.4)	7 (18.4)	2 (28.6)	
Fulani	3 (1.0)	0 (0.0)	2 (3.3)	0 (0.0)	0 (0.0)	0 (0.0)	
Follow-up time (total time at risk, person-months)	2782.6	175.1	544.7	1664.1	330.4	68.3	
Clinical malaria episode (*N* (%))	189 (60.4)	11 (50.0)	37 (60.7)	113 (61.1)	24 (63.2)	4 (57.1)	0.872
Time to first clinical malaria episode (median, months)	10.3	10.2	10.6	10.2	10.5	11.4	0.990

*PM* placental malaria, *SD* standard deviation, *LBW* low birth weight, *ITN* insecticide-treated net, *IQR* interquartile range, *MiP* malaria in pregnancy, *COSMIC* community-based scheduled screening and treatment of malaria in pregnancy: a cluster randomized trial, *IPTp-SP* intermittent preventive treatment during pregnancy with Sulfadoxine-pyrimethamine, *CSST/IPTp-SP* community-based scheduled screening and treatment of malaria in combination with the standard IPTp-SP, *AL* artemether-lumefantrine

### TLR-mediated cytokine responses

**Fig. 2:**
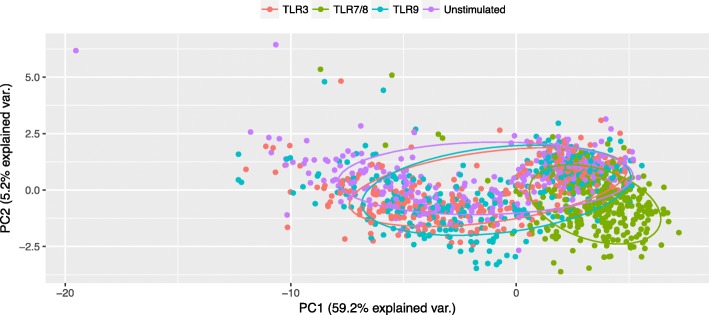
Principal component analysis of cytokine responses to TLR agonists. PCA showing the variance in cytokine responses to the three TLR agonists and unstimulated samples. Ellipses represent the clusters estimated based on principal components 1 and 2

Overall variance of cytokine responses between subjects and stimuli is shown in Fig. 2 by PCA. PC1 and PC2 contribute to explain 59.2% and 5.2% of the variance, respectively. Overall, the responses to TLR3 and TLR9 clustered together with the unstimulated samples in contrast to distinct clustering of TLR7/8 responses, suggesting that TLR3 and TLR9 ligands did not induce—or induced low responses—for most of the analytes. This pattern is further illustrated by log_10_ of ratios of stimulated and unstimulated samples for each TLR agonist (Additional file 1: Figure S1 and S2), which show that few cytokines were produced above the background level following stimulations of TLR3 or TLR9. Compared to unstimulated samples, IP10 was the only analyte significantly induced by the TLR3 agonist (ANOVA, *P* < 0.001), while those significantly induced in response to TLR9 included IFN-α, IL-1RA, MCP-1, and IP-10 (ANOVA, *P* ≤ 0.006). For TLR7/8 stimulation, all analyzed cytokines (with the exception of eotaxin, *P* = 0.319) had a significantly higher concentration than that of unstimulated samples (ANOVA, *P* < 0.05).

### PME and cytokine responses at birth

Variation in cytokine production by PME category is shown as boxplots in Additional file 2: Figure S3–S6. Results indicate that PME modifies innate immune responses to TLR stimulations at different magnitudes, depending on the PME category. The main effect was observed in responses to TLR7/8 stimulation (Additional file 2: Figure S5), being past PM more frequently associated with a significantly higher production of cytokine levels (i.e., IFN-α, IL-2, MIP-1α, RANTES, FGF, G-CSF, GM-CSF) compared with the non-exposed control group (ANOVA, *P* < 0.05). As expected, there was little variation in cytokine levels according to PME category following stimulations by TLR3 or TLR9, as these PRRs ligands induced very low cytokine production. The concentrations of cytokines in unstimulated samples also differed between PME categories. Overall, there was a tendency of lower cytokine levels among infants prenatally exposed to malaria (any category) than in non-exposed infants (Additional file 2: Figure S3). The significant variations were mostly observed with past PM on IL-1β, TNF, IL-7, IL-15, IL-2, IL-4, G-CSF, GM-CSF, HGF, and VEGF and with chronic PM on IL-7, IL-15, IL-2, IFN-γ, IL-17, and GM-CSF (ANOVA, *P* < 0.05). The comparison of biomarker levels in unstimulated samples from the exposed infants did not show a significant difference between PME groups. However, the trend analysis revealed a significant trend towards decreasing production among unstimulated samples from infants born to mothers with peripheral infection to those born to mothers with PM (past, chronic, and acute, respectively) for some biomarkers including IL-10 (*P* for trend = 0.024), IL-12 (*P* for trend = 0.042), and GM-CSF (*P* for trend = 0.032).

Innate immune response to TLR stimulation by PME was further investigated using multivariable linear regression models. The co-factors, besides PME, affecting cytokine responses in each stimulation assessment used in subsequent models are listed in Additional file 3: Table S1. The confounding factors, including gravidity, ethnicity, birth season, LBW, and newborn sex, were controlled for in subsequent models. Results confirmed that following stimulation with TLR7/8 agonist, infants born to mothers with past PM produced a significantly larger breadth of analytes compared to non-exposed individuals (17 cytokines related to all the functional classes analyzed, except anti-inflammatory and Th17-related cytokines) (Table 2). Significant differences were also observed in infants born to mothers with chronic PM (i.e., MIP-1α, MIP-1β, FGF, G-CSF, and GM-CSF) (*P* < 0.05, Table 2). In the case of infants born to mothers with acute PM, only two growth factors (FGF and GM-CSF) had significantly higher mean ratios compared to non-exposed infants, whereas only GM-CSF was higher in infants born to mothers infected during pregnancy but with no PM at delivery. Stimulation of TLR3 resulted in higher IP-10 responses among infants born to mothers with past and chronic PM (*P* = 0.026 and *P* = 0.008, respectively), but lower IL-5 responses (*P* = 0.046 and *P* = 0.033, respectively). Finally, TLR9-mediated FGF and G-CSF responses were found to be significantly higher in infants born to mothers with past PM compared to the control group (*P* = 0.028 and *P* = 0.016, respectively), whereas IL-5 responses were significantly lower in infants born to mothers with chronic PM compared with those in the non-exposed control group (*P* = 0.009).

**Table 2:** Multivariable linear regression analyses assessing the effect of prenatal malaria exposure (PME) categories on TLR-mediated cytokine responses at birth

Cytokines*		Exposed/no PM vs non-exposed		Past PM vs non-exposed		Chronic PM vs non-exposed		Acute PM vs non-exposed	
		Coeff (SE)	*P*	Coeff (SE)	*P*	Coeff (SE)	*P*	Coeff (SE)	*P*
TLR7/8 responses using log_10_ of ratio cytokines concentrations									
Pro	IFN-α	0.15 (0.08)	0.079	*0.19* (*0.08*)	*0.018*	0.09 (0.09)	0.359	0.08 (0.15)	0.591
	IL-1RA	0.15 (0.12)	0.223	*0.22* (*0.11)*	*0.049*	0.15 (0.14)	0.263	0.22 (0.22)	0.308
	IL-1β	0.13 (0.20)	0.522	*0.36* (*0.18)*	*0.049*	0.32 (0.22)	0.140	0.11 (0.35)	0.765
	TNF	0.28 (0.24)	0.238	*0.55* (*0.22)*	*0.013*	0.31 (0.26)	0.229	0.36 (0.42)	0.387
Th1	IL-12	0.18 (0.16)	0.259	*0.34* (*0.14)*	*0.015*	0.30 (0.17)	0.074	0.30 (0.27)	0.276
	IL-2	0.12 (0.07)	0.092	*0.15* (*0.06)*	*0.019*	0.14 (0.08)	0.081	0.09 (0.12)	0.448
	IL-2R	0.16 (0.10)	0.103	*0.23* (*0.09)*	*0.012*	0.19 (0.11)	0.081	0.15 (0.18)	0.398
	IFN-γ	0.18 (0.12)	0.137	*0.29* (*0.11)*	*0.008*	0.25 (0.13)	0.056	− 0.03 (0.21)	0.898
Th2	IL-13	0.14 (0.13)	0.272	*0.25* (*0.12)*	*0.031*	0.25 (0.14)	0.079	0.26 (0.22)	0.237
Chemokines	MIP-1α	0.47 (0.27)	0.085	*0.72* (*0.25)*	*0.004*	*0.59* (*0.30)*	*0.049*	0.57 (0.48)	0.237
	MIP-1β	0.36 (0.25)	0.143	*0.56* (*0.22)*	*0.013*	*0.52* (*0.26)*	*0.049*	0.54 (0.43)	0.214
	RANTES	0.17 (0.10)	0.097	*0.23* (*0.09)*	*0.015*	0.21 (0.11)	0.062	0.07 (0.18)	0.697
Growth factors	EGF	0.10 (0.06)	0.087	*0.12* (*0.05)*	*0.017*	0.09 (0.06)	0.125	0.09 (0.10)	0.360
	FGF	0.16 (0.09)	0.069	*0.22* (*0.08)*	*0.005*	*0.23* (*0.09)*	*0.017*	*0.32* (*0.15)*	*0.037*
	G-CSF	0.18 (0.13)	0.170	*0.35* (*0.12)*	*0.004*	*0.29* (*0.14)*	*0.047*	0.38 (0.23)	0.099
	GM-CSF	*0.34* (*0.16)*	*0.033*	*0.53* (*0.14)*	*< 0.001*	*0.36* (*0.17)*	*0.032*	*0.64* (*0.27)*	*0.020*
	HGF	0.08 (0.06)	0.151	*0.11* (*0.05)*	*0.029*	0.06 (0.06)	0.299	0.04 (0.10)	0.676
TLR9 responses using log_10_ of ratio cytokines concentrations									
Th2	IL-5	− 0.08 (0.07)	0.283	− 0.11 (0.07)	0.008	− *0.20* (*0.08)*	*0.009*	− 0.14 (0.13)	0.267
Growth factors	FGF	0.08 (0.07)	0.231	*0.14* (*0.06)*	*0.028*	0.15 (0.07)	0.050	0.14 (0.12)	0.234
	G-CSF	0.11 (0.08)	0.187	*0.17* (*0.07)*	*0.016*	0.15 (0.09)	0.082	0.09 (0.09)	0.505
TLR3 responses using log_10_ of ratio cytokines concentrations									
Th2	IL-5	− 0.06 (0.06)	0.392	− *0. 12*(*0.06)*	*0.046*	− *0.15* (*0.07)*	*0.033*	− 0.02 (0.11)	0.841
Chemokines	IP-10	0.13 (0.11)	0.222	*0.22* (*0.10)*	*0.026*	*0.32* (*0.12)*	*0.008*	0.10 (0.19)	0.584
Unstimulated samples using log_10_ of crude cytokines concentrations									
Pro	IFN-α	− 0.11 (0.07)	0.154	− *0.14* (*0.07)*	*0.038*	− 0.15 (0.08)	0.069	− 0.01 (0.13)	0.948
	IL-1RA	− 0.24 (0.12)	0.055	− *0.23* (*0.11)*	*0.040*	− 0.23 (0.14)	0.086	− 0.37 (0.21)	0.084
	IL-1β	− 0.35 (0.24)	0.152	− *0.58* (*0.22)*	*0.008*	− 0.50 (0.26)	0.057	− 0.62 (0.42)	0.142
	TNF	− 0.40 (0.26)	0.117	− *0. 61*(*0.23)*	*0.009*	− 0.49 (0.28)	0.079	− 0.47 (0.45)	0.296
Anti	IL-10	− 0.22 (0.32)	0.485	− *0.60* (*0.29)*	*0.041*	− 0.45 (0.35)	0.204	− 0.92 (0.56)	0.105
	IL-7	− *0.34* (*0.10)*	*< 0.001*	− *0.40* (*0.09)*	*< 0.001*	− *0.35* (*0.11)*	*< 0.001*	− *0.40* (*0.17)*	*0.021*
Th1	IL-15	− *0.39* (*0.16)*	*0.015*	− *0.52* (*0.14)*	*< 0.001*	− *0.52* (*0.17)*	*0.003*	− 0.36 (0.28)	0.190
	IL-2	− 0.15 (0.08)	0.064	− *0.16* (*0.07)*	*0.032*	− 0.18 (0.09)	0.053	− 0.12 (0.14)	0.382
	IFN-γ	− 0.14 (0.08)	0.094	− *0.17* (*0.07)*	*0.023*	− *0.21* (*0.09)*	*0.024*	− 0.10 (0.14)	0.494
Th2	IL-13	− *0.25* (*0.12)*	*0.038*	− *0.22* (*0.11)*	*0.042*	− *0.32* (*0.13)*	*0.014*	− 0.12 (0.21)	0.056
	IL-4	− 0.27 (0.14)	0.057	− *0.40* (*0.13)*	*0.002*	− 0.26 (0.15)	0.096	− 0.07 (0.25)	0.769
Th17	IL-17	− *0.13* (*0.07)*	*0.046*	− *0.15* (*0.06)*	*0.016*	− *0.17* (*0.07)*	*0.017*	− 0.18 (0.12)	0.128
Chemokines	MIP-1α	− 0.29 (0.28)	0.297	− *0.56* (*0.25)*	*0.029*	− 0.50 (0.31)	0.102	− 0.37 (0.49)	0.453
	RANTES	− 0.23 (0.12)	0.050	− *0.25* (*0.11)*	*0.023*	− *0.28* (*0.13)*	*0.029*	− 0.19 (0.21)	0.371
Growth factors	EGF	− 0.11 (0.07)	0.106	− *0.12* (*0.06)*	*0.047*	− *0.15* (*0.07)*	*0.049*	− 0.09 (0.12)	0.446
	FGF	− 0.12 (0.08)	0.151	− *0.15* (*0.08)*	*0.049*	− 0. 17(0.09)	0.070	− 0.17 (0.15)	0.244
	G-CSF	− 0.21 (0.17)	0.229	− *0.37* (*0.15)*	*0.019*	− *0.37* (*0.15)*	*0.045*	− 0.33 (0.30)	0.276
	GM-CSF	− 0.36 (0.19)	0.058	− *0.59* (*0.17)*	*< 0.001*	− *0.60* (*0.21)*	*0.004*	− 0.59 (0.33)	0.079
	HGF	− *0.19* (*0.07)*	*0.008*	− *0.20* (*0.06)*	*0.002*	− *0.19* (*0.08)*	*0.015*	− 0.15 (0.12)	0.221
	VEGF	− *0.45* (*0.22)*	*0.041*	− *0.59* (*0.20)*	*0.003*	− 0.43 (0.24)	0.070	− 0.35 (0.38)	0.356

*PM* placental malaria, *Coeff* coefficient, *SE* standard error, *Pro* proinflammatory cytokines, *P p* value, *Anti* anti-inflammatory cytokines, *Th1* Th1-type cytokines, *Th2* Th2-type cytokines, *Th17* Th17, type cytokines. *Only cytokines whose concentrations are significantly modified by categories of PME are presented. Non-exposed category was used as reference in each model. Significant results are shown in italic

Results from the multivariable models confirmed decreased levels of cytokines in unstimulated samples from infants prenatally exposed to malaria compared to the non-exposed control group: lower cytokine responses were found in past PM exposed group (20 cytokines from all the functional classes analyzed), chronic PM (10 cytokines from all the functional classes, except proinflammatory cytokines), acute PM (IL-7 only), and for peripheral infections during pregnancy (IL-7, IL-15, IL-13, IL-17, HGF, VEGF).

### TLR-mediated cytokine responses and risk of clinical malaria during the first year of life

**Fig. 3:**
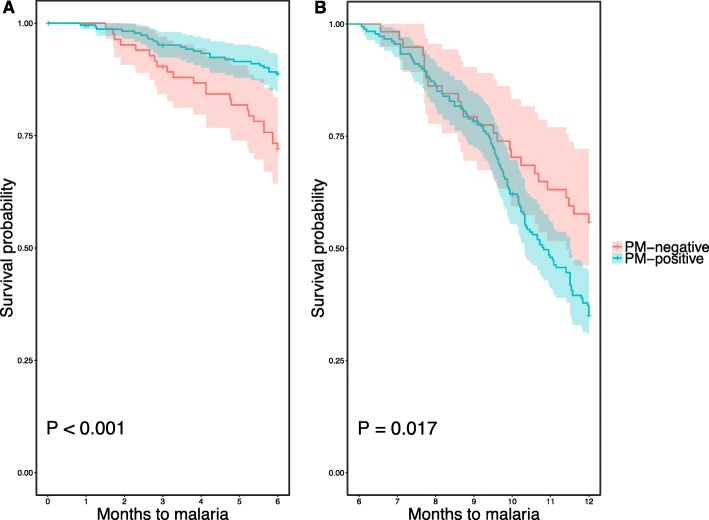
Risk of clinical malaria during the first year of life, by prenatal malaria exposure (placental malaria versus no placental malaria). Kaplan-Meier survival curves (including 95% confidence intervals) stratified by infants born to mothers with (blue line) or without (red line) PM. **a** Clinical malaria episodes during the first 6 months of life. **b** Clinical malaria episodes from 6 to 12 months of life. *P* values were determined by log-rank test

**Fig. 4:**
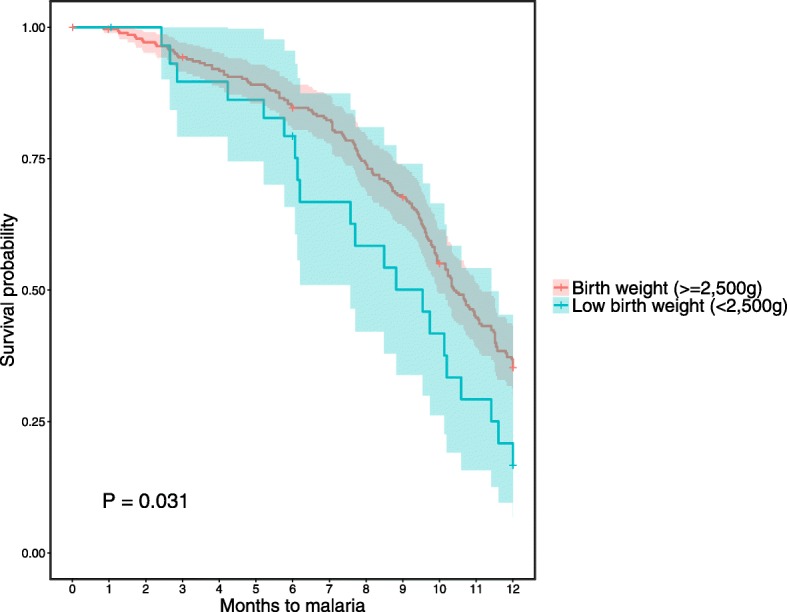
Risk of clinical malaria during the first year of life, by birth weight. Kaplan-Meier survival curves (including 95% confidence intervals) stratified by infants born with a birth weight ≥ 2500 g (red line) and with a birth weight below 2500 g (low birth weight, blue line). *P* value was determined by log-rank test

**Fig. 5:**
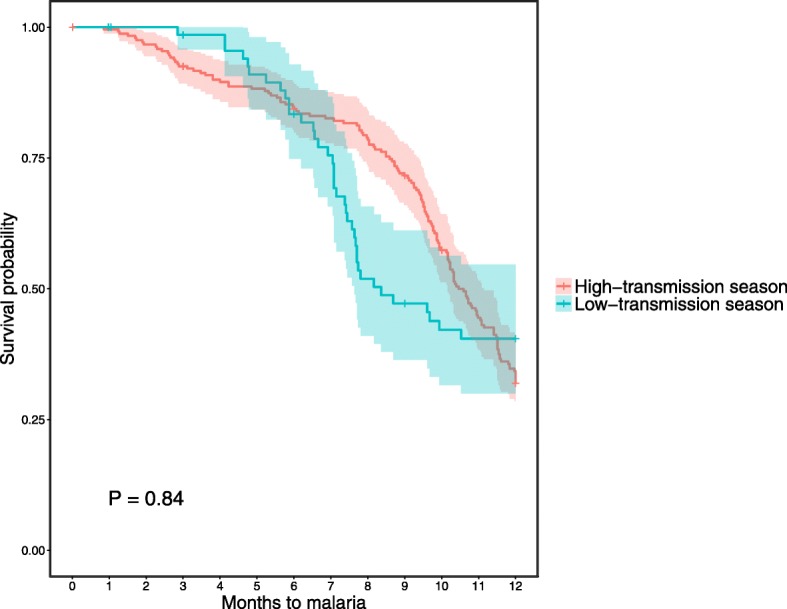
Risk of clinical malaria during the first year of life, by birth season. Kaplan-Meier survival curves (including 95% confidence intervals) stratified by infants born during malaria high-transmission season (July–December, red line) and low-transmission season (January–June, blue line). *P* value was determined by log-rank test

Data on malaria incidence and prevalence among the overall birth cohort has been described elsewhere [39]. In the subgroup of infants included in the present analysis, malaria incidence was 60.4% (189/313) with a median survival time of 10.3 months (Table 1). Among the potential confounding factors analyzed (i.e., gravidity, PME, LBW, birth season, newborn sex, ethnicity, insecticide-treated net (ITN) usage by mothers), PME (Fig. 3) and LBW (Fig. 4) were found to be significantly associated with the risk of clinical malaria and, thus, were included in the Cox multivariable regression analyses. In particular, we found that infants born to mothers with PM had a significantly lower risk of clinical malaria during the first 6 months of life, while they were at higher risk of clinical malaria from age 6 to 12 months, compared to infants born to mothers with no PM. In addition, infants born with LBW had a significant shorter time to first clinical malaria episode than those born with a normal birth weight. Although birth season was not significantly associated with the risk of clinical malaria (Fig. 5), it was included in the models using an interaction term with the timing of clinical malaria to account for differences in the risk of infection between infants due to the high seasonality in malaria transmission in Burkina Faso.

Using crude concentration of cytokines, we found that higher concentrations of eotaxin (in both unstimulated and TLR7/8-stimulated samples), IL-7 (in TLR3-stimulated samples), GM-CSF (in TLR7/8-stimulated samples), and IL-1β (in TLR9-stimulated samples) in the cord blood at birth were significantly associated with an increased risk of subsequent clinical malaria episodes during the first year of life (Table 3). In contrast, an increase in the concentration of IP-10 in TLR3 and TLR9 stimulations was associated with a decreased risk of clinical malaria occurrence in early infancy. When considering biomarkers ratios, increases in TLR3-mediated IL7 response were predictive of an increased risk of clinical malaria attack, while higher TLR9-mediated eotaxin responses and TLR7/8-mediated IL-1RA and IL-12 responses had a protective effect against developing a malaria episode during the first year of life (Table 3). Remarkably, TLR-mediated responses of some biomarkers, which showed a significant prediction of malaria protection/risk during the first 12 months of life (i.e., IL-12 TLR7/8 ratios, IL-1RA TLR7/8 ratios, GM-CSF TLR7/8 crude, IP-10 TLR3 crude), were significantly influenced by in utero exposure to malaria parasites (Additional files 2 and 3) indicating the clinical relevance of the modulation of newborn’s innate immune responses by PME.

**Table 3:** Cox proportional hazards analyses assessing the association between TLR-induced cytokine responses and the risk of malaria during the first year of life. Adjusted hazard ratio and 95% CI for each model is shown

Cytokines**		Unstimulated	TLR3 responses		TLR7/8 responses		TLR9 responses	
		Crude	Crude	Ratios	Crude	Ratios	Crude	Ratios
		AHR (95% CI)	AHR (95% CI)	AHR (95% CI)	AHR (95% CI)	AHR (95% CI)	AHR (95% CI)	AHR (95% CI)
Pro	IFN-α			0.24 (0.05–1.10)				
	IL-1RA*				0.61 (0.36–1.03)	*0.70* (*0.50–0.98*)		
	IL-1β	1.16 (0.98–1.37)	1.15 (0.97–1.36)				*1.18* (*1.01–1.39*)	
	TNF							
Anti	IL-10		1.12 (0.99–1.25)					
	IL-7		*1.80* (*1.26–2.58*)	*1.54* (*1.11–2.15*)				
Th1	IL-15						1.22 (0.96–1.54)	
	IL-12*	1.28 (0.97–1.69)	1.26 (0.95–1.68)	0.27 (0.07–1.15)		*0.76* (*0.59–0.98*)		0.46 (0.20–1.04)
	IL-2							
	IL-2R							
	IFN-γ							
Th2	IL-13							
	IL-5							
	IL-4							
Th17	IL-17							
	IL-6							
Chemokines	IL-8							
	IP-10*	0.64 (0.38–1.08)	*0.66* (*0.46–0.93*)				*0.75* (*0.59–0.96*)	
	MCP-1							
	MIG							0.77 (0.56–1.05)
	MIP-1α						1.13 (0.97–1.31)	
	MIP-1β							
	RANTES							
	EOTAXIN	*1.83* (*1.11–3.01*)	1.60 (0.99–2.58)	0.19 (0.03–1.14)	*1.72* (*1.01–2.94*)			*0.46* (*0.24–0.86*)
Growth factors	EGF				0.29 (0.08–1.07)			
	FGF		1.63 (0.91–2.93)					
	G-CSF	1.24 (0.97–1.57)						
	GM-CSF*				*1.28* (*1.01–1.15*)			
	HGF							
	VEGF							

*HR* hazard ratio, *CI* confidence interval, *Pro* proinflammatory cytokines, *Anti* anti-inflammatory cytokines, *Th1* Th1-type cytokines, *Th2* Th2-type cytokines, *Th17* Th17-type cytokines. **Only results of cox proportional analysis for cytokines with *P* value (*P*) ≤ 0.100 are presented. *Cytokines whose levels were significantly modified by prenatal malaria exposure categories. Significant results are shwon in italic

## Discussion

In this study, we investigated the impact of different manifestations of malaria in pregnancy on both spontaneous and TLR-mediated cytokine production by cord blood cells at birth and we assessed whether these cytokines predicted malaria risk/protection in infancy. Overall, we found that PME has a profound effect on the fetal immune system and that the differential modulation of infants’ innate immune responses by PME could have important implications with regards to malaria susceptibility in infancy. Indeed, we observed that spontaneous cytokine, chemokine, and growth factor production were all significantly lower in samples from exposed versus non-exposed infants. However, following TLR7/8 stimulation, cord blood cells from mothers with past PM (pigment only) were hyper-responsive in comparison to those without evidence of prenatal exposure. Importantly, we identified some responses (both spontaneous and following TLR stimulation) associated with differential malaria risk in infancy.

To our knowledge, this study reports for the first time the effect of these categories of PME on TLR-mediated innate immune responses, as previous studies have focused on the overall effect of PM and/or other types of PME on PRR-mediated cytokines responses [9, 29, 30, 49]. It has been shown that malaria pigment in the placenta is associated with the maturation of cord blood myeloid and plasmocytoid DCs (innate immune cells triggered by TLR7/8 agonists [11, 30]), which may explain why cytokine responses to TLR7/8 stimulation were significantly enhanced in past PM (as well as in chronic PM, although with modest significance possibly due to the smaller sample size) compared to the other PME categories. A number of studies have also explored the effect of in utero malaria exposure on cord blood immune cell populations including dendritic cells (DCs), γδ T cells, CD4^+^ T regulatory, and effector cells [10–15]. Interestingly, all revealed a varying effect of PME categories on cord blood mononuclear cells, thus ultimately demonstrating inter-individual variation in immune responses following different types of PME. Consequently, the differential admixture of cell types across PME categories may be explanatory of the observed differences in cytokine production in the present study. In addition, there is increasing evidence that the innate system has immunological memory [50–54] and that innate stimulations can lead to sensitization to following pathogen exposure, a process termed trained innate immunity [53]. Therefore, in utero exposure could affect TLR responses of cord blood cells through the development of trained immunity.

While TLR7/8 stimulation induced robust cytokine responses, overall cytokine responses induced by TLR3 and TLR9 stimulations were low with limited variations between PME categories, consistent with previous investigations in African children [30, 55, 56] and non-African children [57, 58]. Although TLR3 and TLR9 are endosomal PRRs like TLR7/8, they differ in their responses depending on the cell populations, which may explain differences in their inducible capacity of cytokine responses. Importantly, spontaneous cytokine production by cord blood cells in unstimulated samples also displayed significant variations between PME groups, with a trend towards decreased baseline levels in infants born to mothers with peripheral infection to those born to mothers with PM (past, chronic, and acute PM, respectively). Altogether, our findings are consistent with the hypothesis that PME results in a downregulation of cytokines production that can affect all the important functional classes of cytokines, but followed by a hyper-responsiveness to particular PRR agonists, such as TLR7/8 agonist, as compared to that in non-exposed infants.

Innate immune activation plays a crucial role in host protection as well as pathogenesis during malaria infection [59, 60]. Therefore, the second important aim of our study was to determine the predictive value of cytokines that were significantly influenced by PME for clinical malaria occurrence during the first year of life. Of note, we have shown that PME has a clinical impact on the risk of malaria among the study population. Indeed, we observed that infants born to mothers with PM had a lower risk of clinical malaria during the first 6 months of life, in contrast to that reported in several epidemiological studies [2, 4, 5, 7]. This paradoxal finding may be at least partially explained by the protective effect of maternal antibodies and the strong malaria seasonality of the study area [39], which may make PME dynamics different from other sites. However, we cannot exclude a confounding or explanatory effect of other factors not assessed in this study.

Notably, we observed that some cytokines, which were associated with PME, were independent predictors of malaria risk or protection, demonstrating the clinical relevance of the modulation of infants’ innate immune responses by PME. However, few studies have investigated the predictive value of cytokines, measured at birth either in unstimulated samples [49, 61] or upon stimulation with TLR [30], on the risk of clinical malaria during infancy. Those studies showed a protective prediction of high proinflammatory cytokine levels in unstimulated samples (TNF, TNF-RI, IL-1β), while high levels of anti-inflammatory cytokines such as IL-10 (upon TLR3 and TLR7/8 stimulations) predicted an increased risk of clinical malaria in early childhood [30, 49, 61]. Here, we found that Th1 cytokines and chemokines (IL-12 TLR7/8 ratio and IP-10 TLR3 crude) and cytokines induced upon inflammation (IL-1RA TLR7/8 ratio) were associated with a decreased risk of clinical malaria during the first year of life. These results are in agreement with a key role of IL-12 in the induction of a Th1-type protective immunity against malaria mediated by IFN-γ, TNF, and nitric oxide productions [62–66] and the inhibiting effect on disease severity of IL-1RA on IL-1A and IL-1β (through binding to IL-1 receptors) [67]. For IP-10, a cytokine belonging to the CXC chemokine family that induces chemotaxis, apoptosis, cell growth, and angiostasis, the association with malaria protection observed in this study is in agreement with studies in the murine model [68], although in contrast with others that have shown an association with clinical malaria and disease severity [69–72]. Among biomarkers that were significantly associated with PME, GM-CSF TLR7/8 crude was associated with a risk of developing clinical malaria during the first year of life, which is in contrast with previous observations [73–75], but in agreement with others [76]. These conflicting findings could be related to the fact that cytokines promoting a protective inflammatory environment during malaria infection could become harmful if exaggerated and act in favor of disease manifestation [77–79]. Overall, these results suggest that PME has an impact on malaria risk and that the effect is at least partially mediated by the modulation of TLR and the consequent cytokine responses. Given that past PM, which potentially occurs early during pregnancy, has a profound effect on fetal immune system, a strategy based on screening and treatment of malaria during pregnancy that we proved to benefit infants [40] should be implemented as early as possible during the first trimester.

In this study, two main limitations should be noted. First, some of the PME groups including acute PM and non-exposed groups were small in comparison with others. Therefore, we cannot exclude an underestimation of the effect of acute PM on innate immune responses measured. However, this number reflects the prevalence of PM categories in the main COSMIC trial as most of malaria infections in the placenta were past or chronic PM (95.5%). The relatively limited number of non-exposed controls is due to the high malaria transmission in the study area and the strict definition and recruitment that we applied to this group, where pregnant women had negative RDT/LM and qPCR results at each screening and ANC visit, in addition to negative placental histology. Second, in this study, the measurement of white blood cells population and lymphocyte subsets in cord blood at delivery was not performed while there is evidence that PME can alter myeloid subsets abundance and thus influence TLR-mediated innate immune responses. Therefore, the lack of these information has eventually limited the interpretation of our data.

## Conclusions

In conclusion, despite these limitations, our findings indicate that the various PME categories have different effects on innate immune responses of the newborn at birth, which might drive variation between individuals to malaria susceptibility during the first year of life. The differential alteration of TLR-mediated immune responses by PME categories may have profound implications on immune responses to other infections as well as to vaccines formulated with TLR-based adjuvants in infants prenatally exposed to malaria.

## Additional files

Additional file 1: Boxplots showing variation in cytokine responses by stimulation. **Figure S1.** Boxplots generated using log_10_ of crude concentrations (pg/mL). **Figure S2.** Boxplots generated using log_10_ of ratios (TLR/unstimulated). (PDF 287 kb)

Additional file 2: Changes in cytokine responses according to prenatal malaria exposure categories. **Figure S3.** Boxplots generated using log_10_ of unstimulated crude concentrations (pg/mL). **Figure S4.** Boxplots generated using log_10_ of TLR3/unstimulated ratio. **Figure S5.** Boxplots generated using log_10_ of TLR7/8/unstimulated ratio; **Figure S6.** Boxplots generated using log_10_ of TLR9/unstimulated ratio. Non-expo, non-exposed; expo-no-PM, exposed/no placental malaria; past PM, past placental malaria; chronic-PM, chronic placental malaria; acute PM, acute placental malaria. (PDF 350 kb)

Additional file 3: **Table S1.** Variables included in the linear regression models assessing the effect of prenatal malaria exposure on TLR-mediated cytokine responses at birth. (PDF 88 kb)

## Abbreviations

AL: Artemether-lumefantrine
ANC: Antenatal care
COSMIC: Community-based scheduled screening and treatment of malaria in pregnancy: a cluster randomized trial
CSST/IPTp-SP: Community-based scheduled screening and treatment of malaria in combination with intermittent preventive treatment with sulfadoxine-pyrimethamine
EGF: Epidermal growth factor
FGF: Fibroblast growth factor
G-CSF: Granulocyte-colony stimulating factor
GM-CSF: Granulocyte-macrophage colony stimulating factor
HGF: Hepatocyte growth factor
IFN: Interferon
IL: Interleukin
IP: IFN-γ-inducible protein
IPTp-SP: intermittent preventive treatment during pregnancy with sulfadoxine-pyrimethamine
ITN: Insecticide-treated net
LBW: Low birth weight
LM: Light microscopy
MCP: Monocyte chemottractant protein
MIG: Monokine induced by IFN-γ
MIP: Macrophage inflammatory protein
MiP: malaria in pregnancy
PAMPs: Pathogen-associated-molecular patterns
PM: Placental malaria
PME: Prenatal malaria exposure
PRRs: Pattern recognition receptors
qPCR: Quantitative real-time polymerase chain reaction
RDT: Rapid diagnostic test
TLRs: Toll-like receptors
TNF: Tumor necrosis factor
VEGF: Vascular endothelial growth factor
