# Supplementary figures and images for: Modulation of innate immune responses at birth by prenatal malaria exposure and association with malaria risk during the first year of life
Source: BMC Med. 2018 Nov 2;16:198. doi: 10.1186/s12916-018-1187-3 (PMC6214168; doi:10.1186/s12916-018-1187-3)

Figure S1

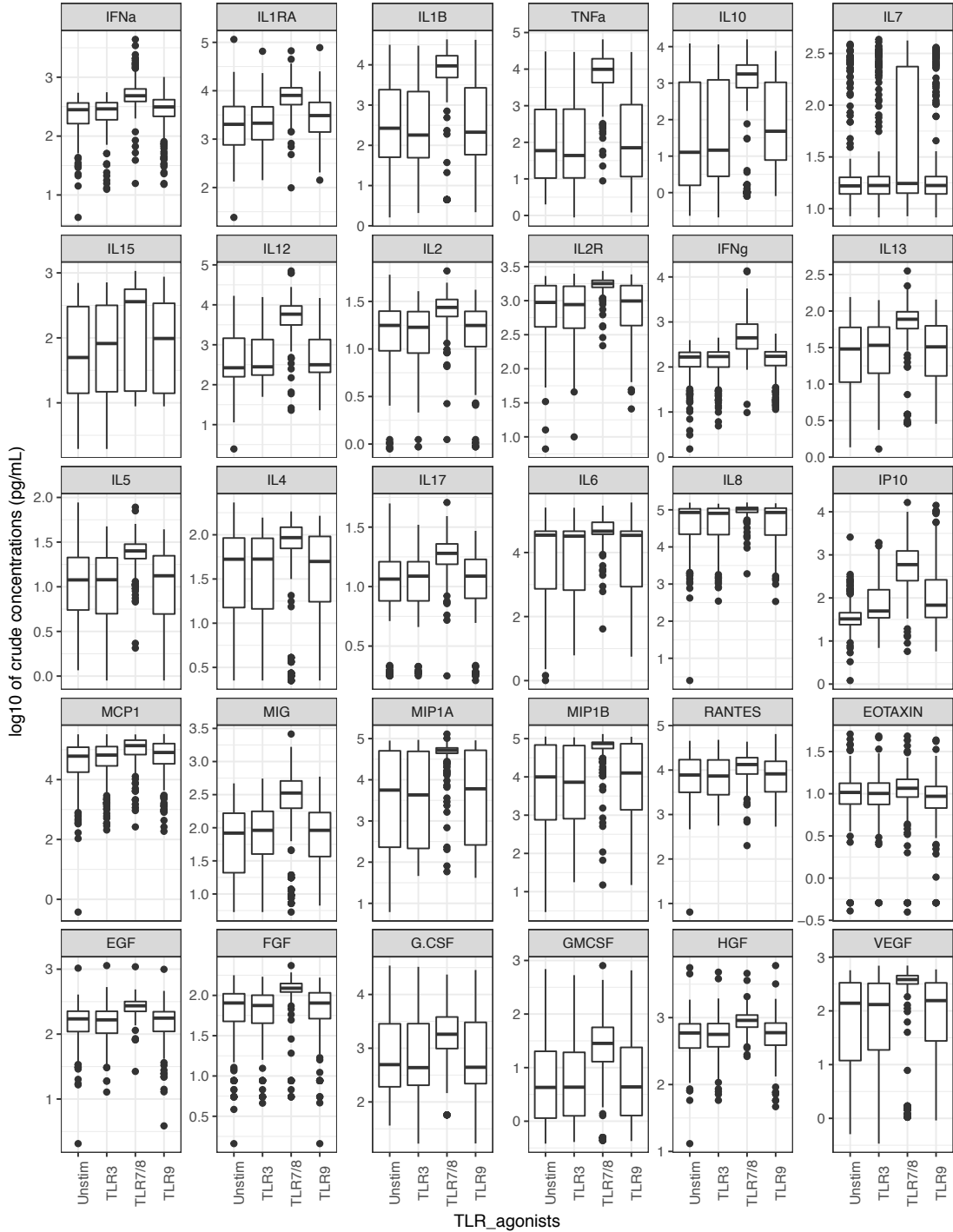

**Figure S2**

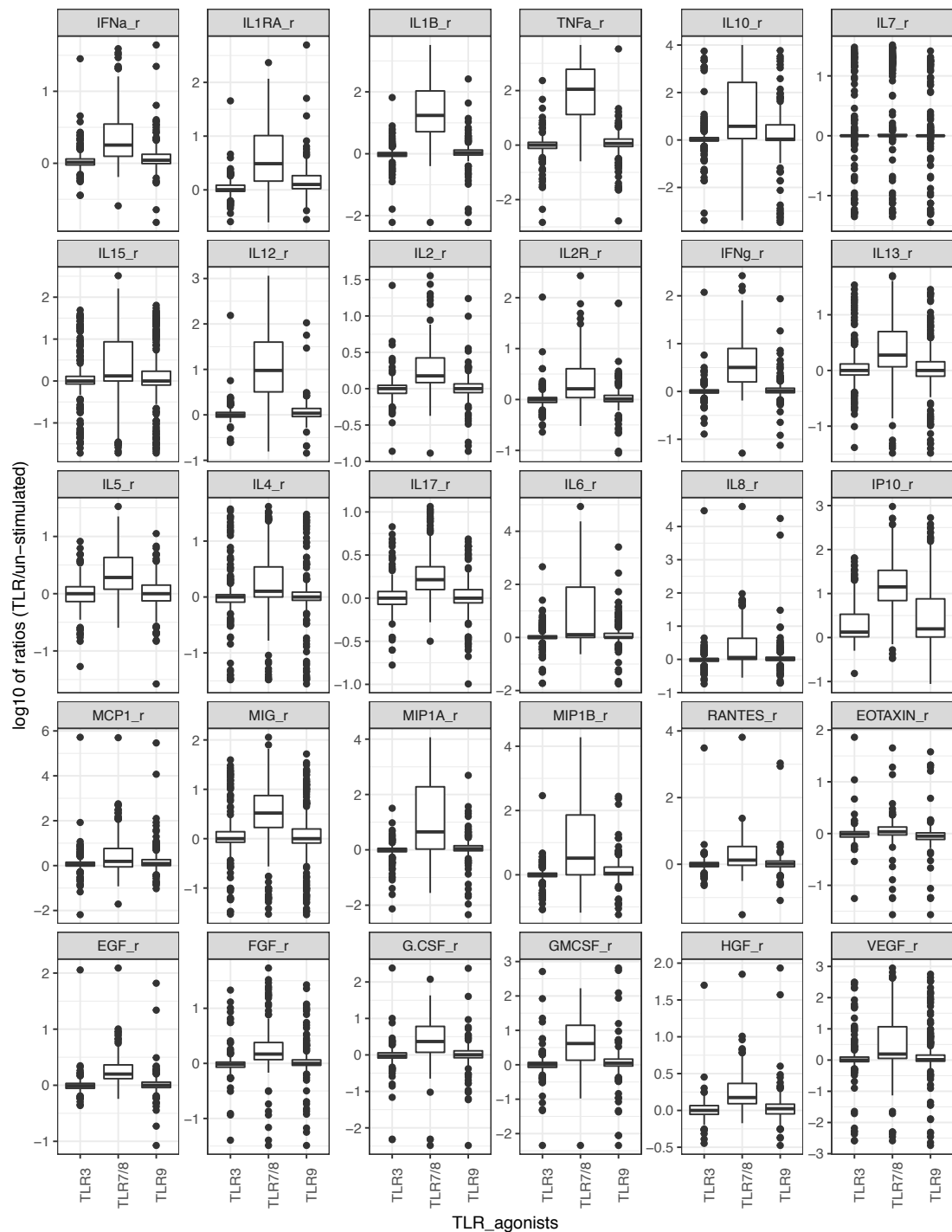

Supplement: Supplementary file 1 — Boxplots showing variation in cytokine responses by stimulation. Figure S1. Boxplots generated using log10 of crude concentrations (pg/mL). Figure S2. Boxplots generated using log10 of ratios (TLR/unstimulated). (PDF 287 kb) [file 12916_2018_1187_MOESM1_ESM.pdf]

Figure S3

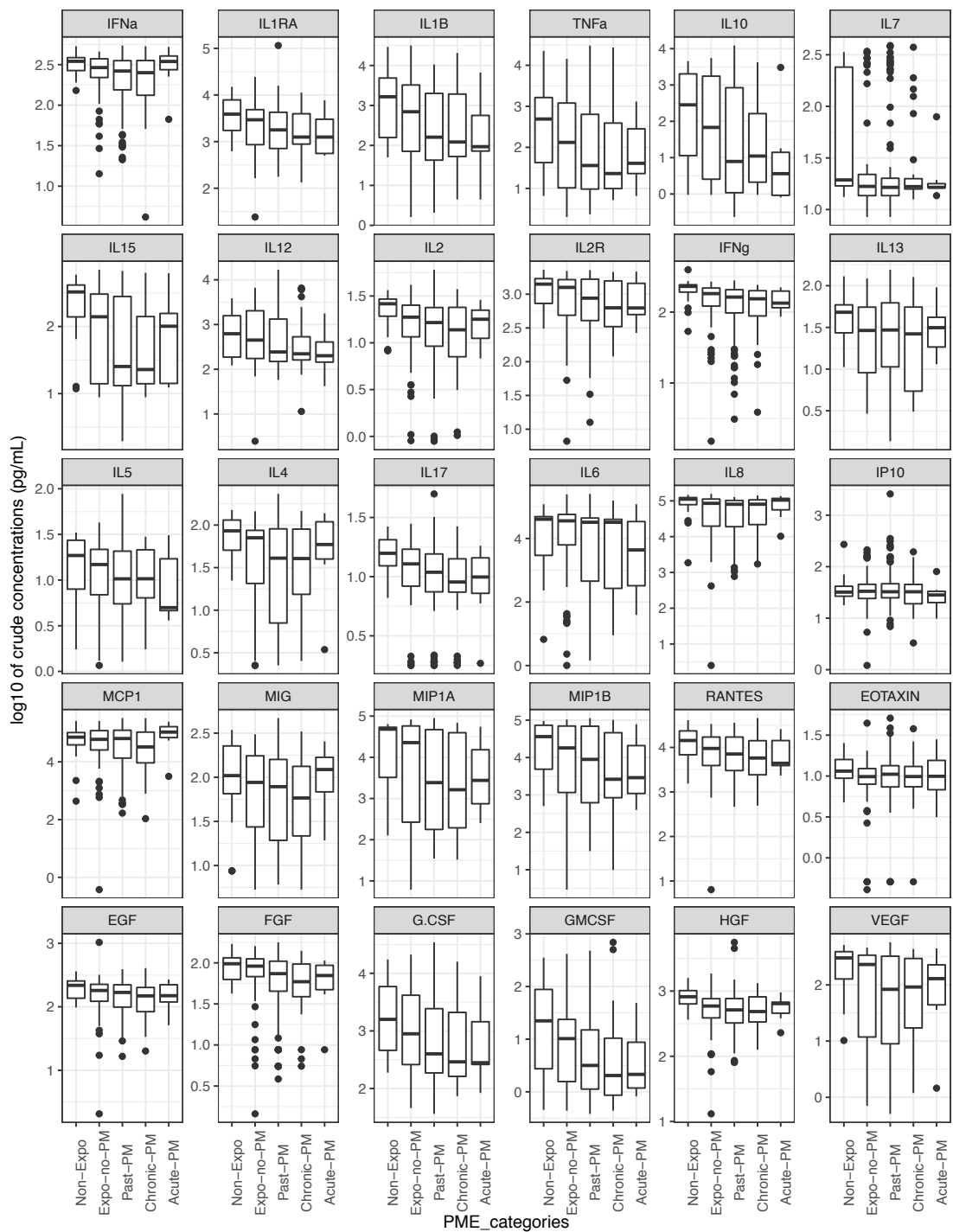

Figure S4

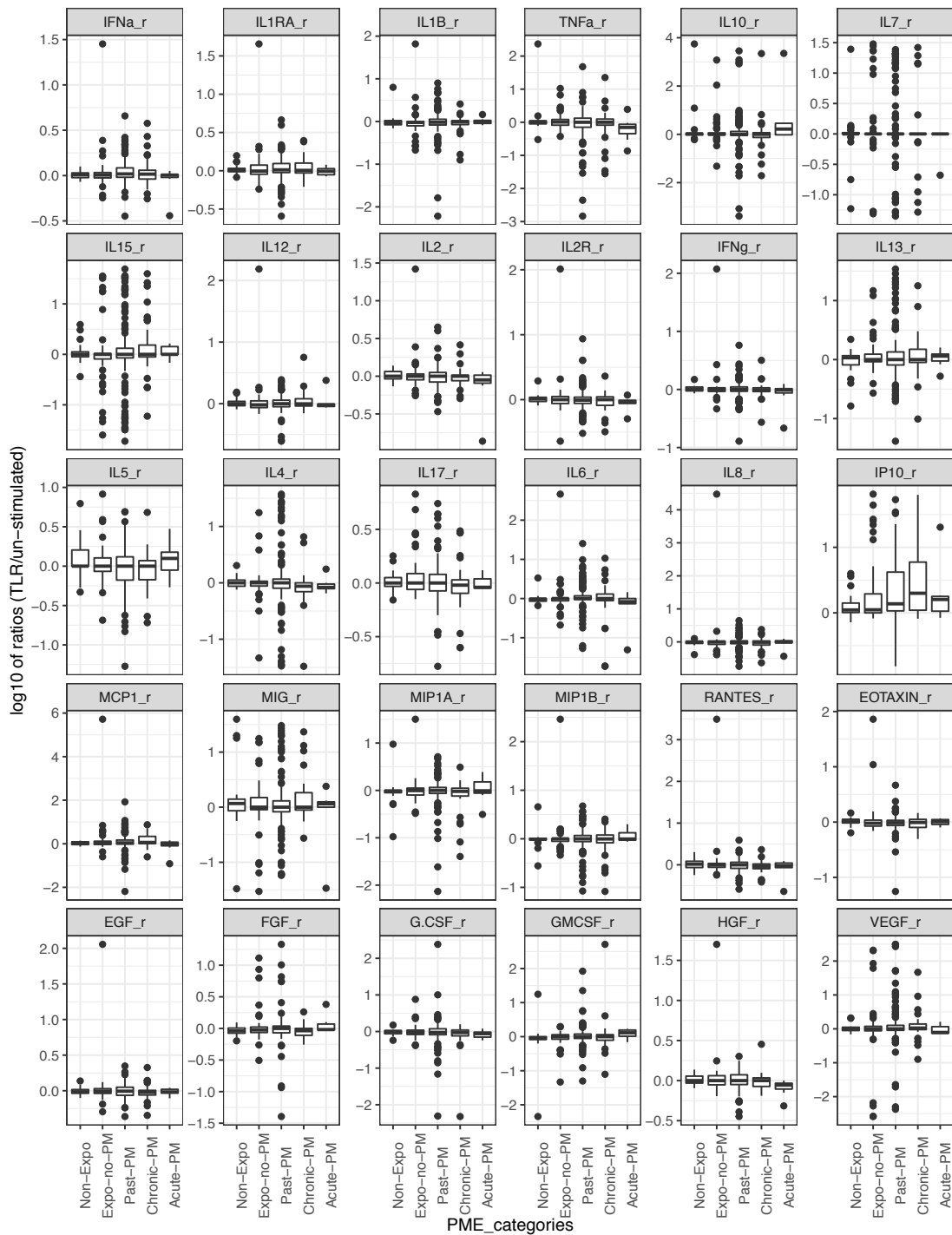

**Figure S5**

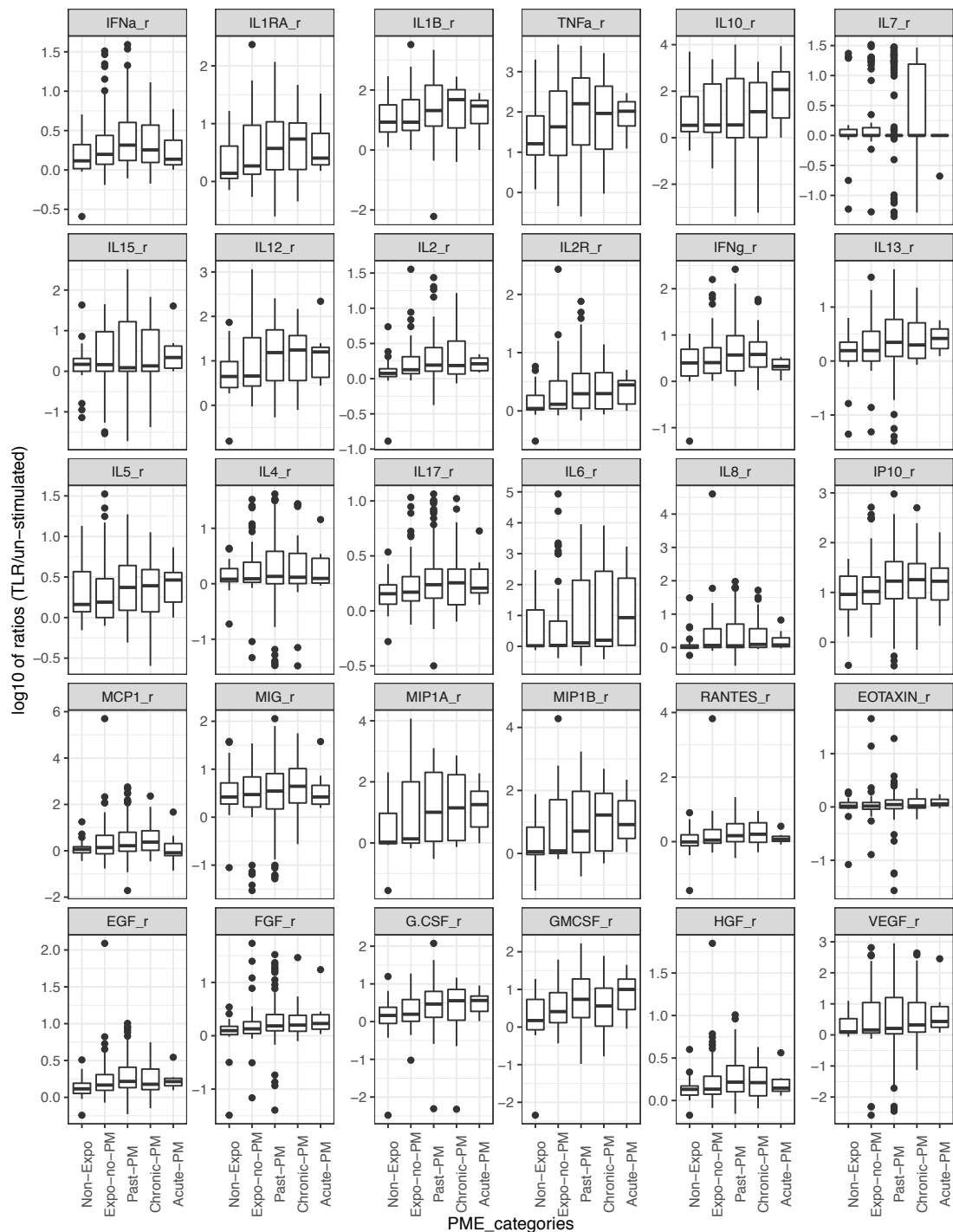

Figure S6

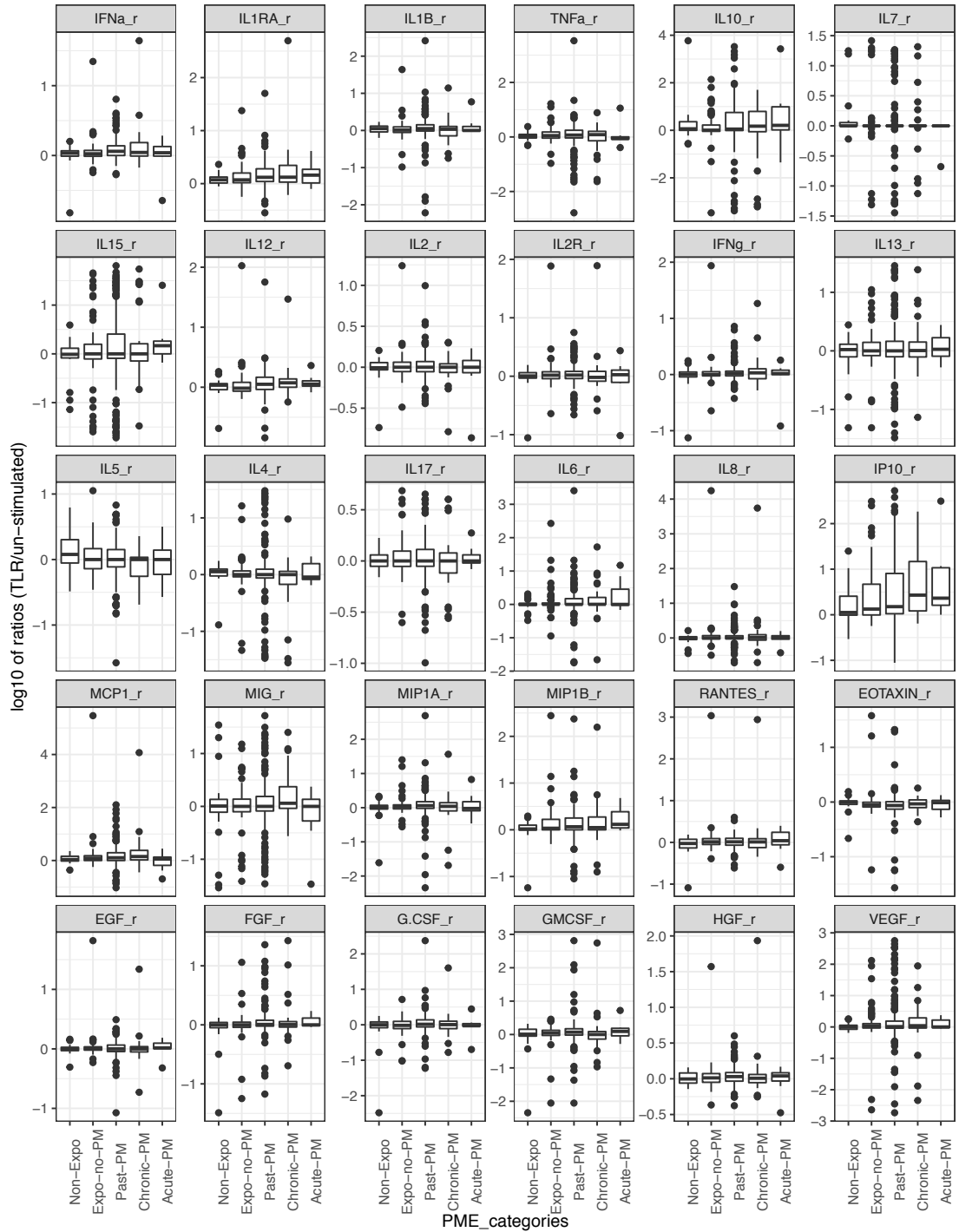

Supplement: Supplementary file 2 — Changes in cytokine responses according to prenatal malaria exposure categories. Figure S3. Boxplots generated using log10 of unstimulated crude concentrations (pg/mL). Figure S4. Boxplots generated using log10 of TLR3/unstimulated ratio. Figure S5. Boxplots generated using log10 of TLR7/8/unstimulated ratio; Figure S6. Boxplots generated using log10 of TLR9/unstimulated ratio. Non-expo, non-exposed; expo-no-PM, exposed/no placental malaria; past PM, past placental malaria; chronic-PM, chronic placental malaria; acute PM, acute placental malaria. (PDF 350 kb) [file 12916_2018_1187_MOESM2_ESM.pdf]
