# Supplementary material for: Modulation of innate immune responses at birth by prenatal malaria exposure and association with malaria risk during the first year of life
Source: BMC Med. 2018 Nov 2;16:198. doi: 10.1186/s12916-018-1187-3 (PMC6214168; doi:10.1186/s12916-018-1187-3)
Supplement: Supplementary file 3 — Table S1. Variables included in the linear regression models assessing the effect of prenatal malaria exposure on TLR-mediated cytokine responses at birth. (PDF 88 kb) [file 12916_2018_1187_MOESM3_ESM.pdf]

Table S1

Cytokines		Variables included in the models			
		Un-stimulated	TLR3 (ratios)	TLR7/8 (ratios)	TLR9 (ratios)
Pro-inflammatory	IFN-α	PME, birth season, sex	None	PME, birth season, gravidity, sex	Birth season, sex
	IL-1RA	PME, gravidity	None	PME, birth season, gravidity	Birth season, gravidity
	IL-1β	PME, birth season	None	PME, Ethnicity	LBW
	TNF-α	PME, birth season	None	PME, birth season	Birth season
Anti-inflammatory	IL-10	PME, birth season	None	Ethnicity, sex	None
	IL-7	PME	None	None	Gravidity
Th1-type	IL-15	PME, birth season	None	None	None
	IL-12	Birth season	None	PME, birth season	Birth season
	IL-2	PME, birth season, gravidity, sex	None	PME, birth season, gravidity, sex	None
	IL-2R	Birth season, sex	None	PME, birth season	None
	IFN-λ	PME, birth season, sex	None	PME	None
Th2-type	IL-13	PME	None	PME, gravidity	Gravidity
	IL-5	Birth season	PME, sex	None	PME
	IL-4	PME, birth season	None	None	None
Th17-type	IL-17	PME, birth season	None	PME, birth season	None
	IL-6	Birth season	None	Birth season	None
Chemokines	IL-8	Birth season	None	None	None
	IP10	Birth season, gravidity	PME, birth season	Birth season, gravidity	Birth season, gravidity
	MCP-1	None	None	Birth season, gravidity	None
	MIG	Birth season	None	None	None
	MIP-1α	PME, birth season	None	PME, birth season	Birth season
	MIP-1β	Birth season	None	PME, birth season	Birth season
	RANTES	PME, birth season	None	PME, birth season, sex	None
	EOTAXIN	Birth season	Gravidity	Gravidity	Gravidity
Growth factors	EGF	PME, birth season	None	PME	None
	FGF	PME, birth season	None	PME	PME
	G-CSF	PME, birth season	None	PME	PME
	GM-CSF	PME, birth season	None	PME	None
	HGF	PME	None	PME, birth season	None
	VEGF	PME, birth season	None	None	None

PME, prenatal malaria exposure.
